# Stochastic Gradient Annealed Importance Sampling for Efficient Online Marginal Likelihood Estimation †

**DOI:** 10.3390/e21111109

**Published:** 2019-11-12

**Authors:** Scott A. Cameron, Hans C. Eggers, Steve Kroon

**Affiliations:** 1 Department of Physics, Stellenbosch University, Stellenbosch 7600, South Africa; 2 National Institute for Theoretical Physics, Stellenbosch 7600, South Africa; 3 Computer Science Division, Stellenbosch University, Stellenbosch 7600, South Africa

**Keywords:** marginal likelihood, evidence, nested sampling, annealed importance sampling, Monte Carlo, stochastic gradients, SGHMC

## Abstract

We consider estimating the marginal likelihood in settings with independent and identically distributed (i.i.d.) data. We propose estimating the predictive distributions in a sequential factorization of the marginal likelihood in such settings by using stochastic gradient Markov Chain Monte Carlo techniques. This approach is far more efficient than traditional marginal likelihood estimation techniques such as nested sampling and annealed importance sampling due to its use of mini-batches to approximate the likelihood. Stability of the estimates is provided by an adaptive annealing schedule. The resulting stochastic gradient annealed importance sampling (SGAIS) technique, which is the key contribution of our paper, enables us to estimate the marginal likelihood of a number of models considerably faster than traditional approaches, with no noticeable loss of accuracy. An important benefit of our approach is that the marginal likelihood is calculated in an online fashion as data becomes available, allowing the estimates to be used for applications such as online weighted model combination.

## 1. Introduction

Marginal likelihood (ML), sometimes called evidence, is a quantitative measure of how well a model can describe a particular data set; it is the probability that the data set occurred within that model. Consider a Bayesian model with parameters θ for a data set $D={\left\{y_{n}\right\}}_{n=1}^{N}$. The ML is the integral

Z:=p(D)=∫p(D|θ)p(θ)dθ,

where p(D|θ) is the likelihood and p(θ) is the prior. In this paper, we consider the case where the data are conditionally independent given the parameters $p\left(D|\theta \right)=\prod_{n}p\left(y_{n}|\theta \right)$, as is common in many parametric models. This restriction is to ensure that a central limit theorem applies to the stochastic likelihood approximation. This can be weakened to any factorization that exhibits a central limit theorem, such as conditionally Markov data and autoregressive models. The posterior distribution over a set of models is proportional to their MLs, and so approximations to ML are sometimes used for model comparison and weighted model averaging [1] (chapter 12). The above integral is typically analytically intractable for any but the simplest models, so one must resort to numerical approximation methods.

Nested sampling (NS) [2] and annealed importance sampling (AIS) [3] are two algorithms able to produce accurate estimates of the ML. NS accomplishes this by sampling from the prior under constraints of increasing likelihood and AIS by sampling from a temperature annealed distribution $\propto \;p{\left(D|\theta \right)}^{\lambda }p\left(\theta \right)$ and averaging over samples with appropriately calculated importance weights. Although NS and AIS produce accurate estimates of the ML, they tend to scale poorly to large data sets due to the fact that they need to repeatedly calculate the likelihood function: for NS, this is to ensure staying within the constrained likelihood contour; for AIS, the likelihood must be calculated both to sample from the annealed distributions using some Markov chain Monte Carlo (MCMC) method as well as to calculate the importance weights. Calculation of the likelihood is computationally expensive on large data sets. To combat this problem, various optimization and sampling algorithms instead use stochastic approximations of the likelihood by sub-sampling the data set into mini-batches [4].

Unfortunately, NS and AIS cannot trivially use mini-batching in their vanilla form to improve scalability. Using stochastic likelihood approximations changes the statistics of the likelihood contours in NS, allowing particles to occasionally move to lower likelihood instead of higher, violating the basic assumptions of the algorithm. Vanilla AIS could benefit from using stochastic likelihood gradients during the MCMC steps, but introducing stochasticity into the importance weights would bias the results.

The key contributions of this work are as follows:

- We introduce stochastic gradient annealed importance sampling (SGAIS), which combines stochastic gradient MCMC with annealed importance sampling to estimate the ML in an online fashion using mini-batch Bayesian updating.
- SGAIS enables efficient ML estimation for streaming data and for large data sets, which was not previously feasible.
- We illustrate how SGAIS can be used to identify distribution shift in the data when applied in an online setting.
- We empirically analyze the behavior of SGAIS and its robustness to various choices of algorithm parameters.

We illustrate our approach by calculating ML estimates on simulated data generated with three simple models. For these models, we obtain considerable speedup over nested sampling and annealed importance sampling on data sets with one million observations, without noticeable loss in accuracy.

## 2. Sequential Marginal Likelihood Estimation

The ML can be factorized, through the product rule, into a product of predictive distributions $Z=\prod_{n}p\left(y_{n}|y_{<n}\right)$, where

(1) $$p\left(y_{n}|y_{<n}\right)=\int p\left(y_{n}|\theta \right)p\left(\theta |y_{<n}\right)\;d\theta .$$

Throughout this manuscript, we will present our approach as Bayesian updating based on one observation at a time. We do this for notational simplicity, with the understanding that the extension to Bayesian updating with multiple observations is mathematically trivial. In our experiments, Section 6, we decompose the data into chunks of a fixed size, rather than one data point at a time. Assuming one is able to produce accurate estimates $\hat{p}\left(y_{n}|y_{<n}\right)$ of the predictive probabilities, the log-ML can be approximated by $\log\hat{Z}=\sum_{n}\log\hat{p}\left(y_{n}|y_{<n}\right)$. In this way, the difficult problem of estimating an integral of an extremely peaked function, p(D|θ), reduces to the easier problem of estimating many integrals of smoother functions $p(y_{n}|\theta )$.

Many sequential Monte Carlo (SMC) methods use a similar approach and calculate predictive estimates using a combination of importance resampling and MCMC mutation steps [5]. Generic examples of such algorithms are the bootstrap particle filter [6], which is often used for posterior inference in hidden Markov models and other latent variable sequence models [5], and the “left-to-right” algorithm, which is used in [7] to evaluate topic models.

The computational efficiency of using this approach depends on the method of approximating Equation (1). In previous work [8], we proposed the estimator $\hat{p}\left(y_{n}|y_{<n}\right)=\frac{1}{M}\sum_{i=1}^{M}p\left(y_{n}|\theta_{i}\right)$, where each $\theta_{i}$ is drawn from the posterior distribution $p(\theta |y_{<n})$ using MCMC methods. This paper extends this idea by combining Bayesian updating with annealing, which we describe in Section 3. Since samples from the previous posterior, $p(\theta |y_{<n-1})$, would generally be available at each step, we expect that only a small number of steps will be needed to accurately sample from the next posterior distribution, $p(\theta |y_{<n})$. Metropolis–Hastings-based MCMC algorithms would have to iterate over all previous n−1 data points in order to calculate the acceptance probability for each Markov transition, and so using them to estimate logZ in this sequential manner would scale at least quadratically in *N*. The key computational improvement in our approach comprises the use of stochastic gradient-based MCMC algorithms such as the stochastic gradient Hamiltonian Monte Carlo (SGHMC) [9]. SGHMC utilizes mini-batching, allowing one to efficiently draw samples from the posterior distribution $p(\theta |y_{<n})$ even when *n* is large. We call this approach stochastic gradient annealed importance sampling (SGAIS).

## 3. Bayesian Updating with Annealing

Annealed importance sampling (AIS) [3] is a Monte Carlo method that generates samples from a sequence of intermediate distributions, bridging from a simple distribution that can be sampled easily (usually the prior) to a desired distribution (usually the posterior). AIS also produces an unbiased estimate of the normalizing constant (the ML) for the desired distribution as a byproduct. The sequence of distributions is typically chosen to have the form $f_{t}\left(\theta \right)=p{\left(D|\theta \right)}^{\lambda_{t}}p\left(\theta \right)$, where 0 = $\lambda_{0}<\lambda_{1}<\ldots <\lambda_{T}=1$. At time step t=0, a number of particles are drawn from the prior, and at each subsequent time step t=1,…,T, an MCMC operator that leaves the distribution $f_{t}$ invariant is applied to each particle. The importance weights are initialized as the normalizing constant of the prior, which is typically 1, and at each time step are updated as follows:

$$w_{i}^{\left(t\right)}\leftarrow w_{i}^{\left(t-1\right)}\frac{f_{t}\left(\theta_{i}^{\left(t-1\right)}\right)}{f_{t-1}\left(\theta_{i}^{\left(t-1\right)}\right)}=w_{i}^{\left(t-1\right)}\;p{\left(D|\theta_{i}^{\left(t-1\right)}\right)}^{\lambda_{t}-\lambda_{t-1}}\;i=1,\ldots ,M,$$

where $\theta_{i}^{\left(t\right)}$ is the *i*^th^ particle at time step *t*. For any function *h*, the unnormalized posterior expectation

$$\int h\left(\theta \right)f_{T}\left(\theta \right)\;d\theta =\int h\left(\theta \right)p\left(D|\theta \right)p\left(\theta \right)\;d\theta$$

can be estimated using *M* particles by $\frac{1}{M}\sum_{i}h\left(\theta_{i}^{\left(T-1\right)}\right)w_{i}^{\left(T\right)}$. This estimator $\hat{H}$ is unbiased and corresponds to the ML in the case h(θ)=1 and the product of the ML and the posterior predictive $p(y^{\prime }|D)$ in the case $h\left(\theta \right)=p\left(y^{\prime }|\theta \right)$. Although it is possible to use stochastic gradient-based MCMC algorithms to update the particles in this setting, the importance weight updates would normally still require iterating over the whole data set at each time step. To circumvent this, we propose using AIS sequentially to calculate predictive probabilities in a Bayesian updating setting, as described in Section 2, instead of on the full data set. This is equivalent to using the following sequence of intermediate distributions:

(2) $$f_{n}^{\left(t\right)}\left(\theta \right)=p{\left(y_{n}|\theta \right)}^{\lambda_{t}}\left[\prod_{k<n}p\left(y_{k}|\theta \right)\right]p\left(\theta \right).$$

In principle, λ may now depend on both *n* and *t*. Since we will choose the annealing schedule adaptively anyway, this notation is suppressed. From an SMC perspective, this can be considered a combination of thermal and data tempering [5]. The corresponding importance weights can then be calculated without iterating over the entire data set;

$$w_{i}^{\left(t\right)}\leftarrow w_{i}^{\left(t-1\right)}\frac{f_{n}^{\left(t\right)}\left(\theta_{i}^{\left(t-1\right)}\right)}{f_{n}^{\left(t-1\right)}\left(\theta_{i}^{\left(t-1\right)}\right)}=w_{i}^{\left(t-1\right)}p{\left(y_{n}|\theta_{i}^{\left(t-1\right)}\right)}^{\lambda_{t}-\lambda_{t-1}}.$$

This result is of central importance to this paper: only by using AIS within a Bayesian updating setting can we effectively take advantage of mini-batching and stochastic gradients.

So far, the choice of each $\lambda_{t}$ is not specified. While any increasing sequence of values for $\lambda_{t}$ (annealing schedule) guarantees an unbiased estimator of the ML, a poor choice can result in high variance of the ML estimator. Grosse et al. [10] point out that a linear sequence typically results in poor performance, and instead, they recommend a sigmoidal annealing schedule, $\lambda_{t}=\sigma \left(\delta \left(2t/T-1\right)\right)$ for some δ. We expect that during early Bayesian updating steps, i.e., when *n* is small, the relative prior $p(\theta |y_{<n-1})$ and posterior $p(\theta |y_{<n})$ may differ substantially, and so many intermediate distributions may be needed. However, once *n* becomes large, the relative prior and posterior will likely be similar and only a few intermediate distributions should be required. For this reason, it is preferable that the annealing schedule be chosen adaptively. An adaptive annealing schedule can be chosen by specifying a target effective sample size (ESS) [11] and choosing the next λ such that the ESS is approximately equal to this target [12,13]. For the choice of intermediate distributions given in Equation (2), the ESS used for the weight update is

$$\mathrm{ESS}\left(\Delta \right):=\frac{{\left(\sum_{i}\omega_{i}\left(\Delta \right)\right)}^{2}}{\sum_{i}\omega_{i}{\left(\Delta \right)}^{2}},$$

where $\Delta :=\lambda_{t}-\lambda_{t-1}$ and

$$\omega_{i}\left(\Delta \right):=\frac{f_{n}^{\left(t\right)}\left(\theta_{i}^{\left(t-1\right)}\right)}{f_{n}^{\left(t-1\right)}\left(\theta_{i}^{\left(t-1\right)}\right)}=p{\left(y_{n}|\theta_{i}^{\left(t-1\right)}\right)}^{\Delta }.$$

Our proposed method combining sequential Bayesian updating and AIS is outlined in Algorithm 1. As in other SMC algorithms, one can optionally resample the particles $\theta_{i}$ proportionally to their importance weights $w_{i}$ before performing each MCMC update on line 9, thereafter setting $w_{i}\;\leftarrow \;\frac{1}{M}\sum_{i}w_{i}$. Resampling can help to reduce variance in the estimator but can cause mode collapse if few particles are used. See [5,12] for more details on resampling. After each annealing sequence is completed (i.e., after the completion of the inner while-loop in the algorithm), one can estimate the ML of the data observed so far by $\hat{p}\left(y_{\leq n}\right):=\frac{1}{M}\sum_{i}w_{i}$.

**Algorithm 1** Stochastic Gradient Annealed Importance Sampling
**Input:** Data $D={\left\{y_{n}\right\}}_{n=1}^{N}$, number of particles *M*, pdfs p(y|θ),p(θ), target ESS: ESS^*^
**Output:** $\hat{Z}$ estimator of marginal likelihood
1:$\forall_{i}$: sample $\theta_{i}∼p\left(\theta \right)$2:$\forall_{i}$: $w_{i}\leftarrow 1$3:**for** n=1,…,N **do**
4:    λ←05:    **while** λ<1 **do**6:        $\Delta \leftarrow {\mathrm{argmin}}_{\Delta }\left[\mathrm{ESS}\left(\Delta \right)-{\mathrm{ESS}}^{*}\right]$             ▹Δ∈(0,1−λ]7:        λ←λ+Δ8:        $\forall_{i}$: $w_{i}\leftarrow w_{i}p{\left(y_{n}|\theta_{i}\right)}^{\Delta }$9:        $\forall_{i}$: $\theta_{i}\leftarrow \mathrm{SGHMC}\left(\theta_{i},{\hat{U}}_{n}^{\left(\lambda \right)}\right)$   ▹ potential energy ${\hat{U}}_{n}^{\left(\lambda \right)}$ defined in Equation (4)10:    **end while**11:
**end for**
12:
**return** $\hat{Z}=\frac{1}{M}\sum_{i}w_{i}$



## 4. Stochastic Gradient Hamiltonian Monte Carlo

SGHMC [9] generically simulates a Brownian particle in a potential U(θ) by numerically integrating the Langevin equation

$$\begin{array}{cc} d\theta & =v\;dt,\\ dv & =-\nabla U\left(\theta \right)\;dt-\gamma v\;dt+\sqrt{2\gamma }\;dW, \end{array}$$

where γ is the friction coefficient and *W* is the standard Wiener process. See [14] (chapter 5) for an introduction to stochastic differential equations. It can be shown through the use of a Fokker–Planck equation [15] that the above dynamics converge to the stationary distribution $p\left(\theta ,v\right)\propto \exp(-U\left(\theta \right)-\frac{1}{2}v^{2})$. We can use this to sample from the full data posterior by using a potential energy equal to the negative log joint, U(θ):=−logp(D,θ). The numeric integration is typically discretized [9,16] as

(3) $$\begin{array}{cc} \Delta \theta & =v,\\ \Delta v & =-\eta \nabla \hat{U}\left(\theta \right)-\alpha v+\epsilon \sqrt{2(\alpha -\hat{\beta })\eta }, \end{array}$$

where η is called the learning rate, 1−α is the momentum decay, ϵ is a standard Gaussian random vector, $\hat{\beta }$ is an optional parameter to offset the variance of the stochastic gradient term, and $\hat{U}\left(\theta \right)$ is an unbiased estimate of U(θ) calculated on independently sampled mini-batches. Since U(θ) grows with the size of the data set, a small learning rate $\eta ∼O\left(\frac{1}{\left|D\right|}\right)$ is required to minimize the discretization error. The variance of the stochastic gradient term is proportional to $\eta^{2}$, while the variance of the injected noise is proportional to η, so in the limit η→0, stochasticity in the gradient estimates becomes negligible and the correct continuum dynamics are recovered, even if one ignores the errors from the stochastic gradient noise by using $\hat{\beta }=0$. We refer the reader to [9,17,18] for an in-depth analysis of the algorithm parameters.

For the purposes of Bayesian updating and annealing, we use SGHMC with a potential energy that leaves the distribution $f_{n}^{\left(t\right)}$ in Equation (2) invariant, $U\left(\theta \right)=-\log f_{n}^{\left(t\right)}\left(\theta \right)$. We can approximate this potential energy stochastically by

(4) $${\hat{U}}_{n}^{\left(\lambda \right)}\left(\theta \right)=-\lambda \log p\left(y_{n}|\theta \right)-\frac{n-1}{\left|B\right|}\sum_{y\in B}\log p\left(y|\theta \right)-\log p(\theta ),$$

where the mini-batch is drawn independently and identically distributed (i.i.d.) with replacement from the set of all previous data points, i.e., $B\subset \{y_{k}|k<n\}$. Since ${\hat{U}}_{n}^{\left(\lambda \right)}\left(\theta \right)$ is an unbiased estimator of $-\log f_{n}^{\left(t\right)}\left(\theta \right)$ with finite variance (assuming the model assigns a non-zero probability to all observations), SGHMC with this potential energy will leave $f_{n}^{\left(t\right)}$ invariant in the limit η→0.

## 5. Online Marginal Likelihood Estimation

Our proposed approach is particularly efficient for ML estimation in applications in which new data are continually becoming available, since the marginal cost to update the ML estimate based on new data is independent of the amount of previously processed data. Some examples of such applications are: finance modeling, monitoring audio or video or other sensor readings, applications in security, process control, etc. In these types of applications, vanilla NS or AIS would have to recalculate the ML from scratch, without leveraging the previous ML estimates.

One challenge with estimating ML online using SGAIS is the generation of mini-batches that are uniformly sampled from historical data. This challenge can be tackled with the help of reservoir sampling. If a large enough reservoir is kept to be representative of the previously processed data, mini-batches could be drawn uniformly from the reservoir instead [19]. If the data set is too large to fit on disk or is only available during streaming, then this approach makes it possible to estimate ML, whereas using NS or AIS to estimate ML would not even be possible.

In online scenarios, the data may also exhibit various degrees of non-stationarity. This may happen due to a shift in the underlying data-generating distribution. Such distribution shifts would typically cause a noticeable change in the ML, which can then be used for change-point detection.

## 6. Experiments

### 6.1. Experimental Setup

To evaluate the accuracy and run-time performance of our proposed approach, we estimate the ML for three simple models on simulated data sets in an online fashion. We further evaluate the robustness of SGAIS under various choices of algorithm parameter values.

**Default Parameters.** We use mini-batches of size 500, with the following SGHMC parameters: η=0.1/N, α=0.2, and $\hat{\beta }=0$. Predictive distributions are approximated using M=10 particles and 20 burn-in steps for each intermediate distribution. We use a target ESS of 5 for adaptive annealing. As mentioned in Section 2, rather than Bayesian updating by adding a single observation at a time, we add chunks of data at a time that are the same size as the mini-batches. These parameter choices are not necessarily the optimal choice for the models below. Instead, we chose the SGHMC parameters based approximately on those suggested in [9], and we chose the number of particles and target ESS to hopefully be sufficient for adaptive annealing but small enough to result in a short running time.

**NS and AIS.** As our reference standards of accuracy, we implemented NS and AIS. Both NS and AIS implementations use SGHMC as their MCMC kernel. For NS, this is to sample from the prior; for AIS, this is to sample from the intermediate annealed distributions. For AIS, we use the same parameters as for our sequential sampler, except that each MCMC step uses the whole data set instead of mini-batches. We implement NS with 20 SGHMC steps to sample from the constrained prior. NS still requires the full data set to check the constraints and so cannot take advantage of mini-batching. For SGHMC used with NS, we used parameters $\eta =10^{-3}$, α=0.1, and $\hat{\beta }=0$ because there is no gradient noise when sampling from the prior. Results reported are for two particles; more behave similarly but are slower. We allow NS to run until the additive terms are less than 1% of the current $\hat{Z}$ estimate. This is a popular stopping criterion and is also used in [10]. More information about our implementations is given in Appendix A.

**Sensitivity Analysis.** We investigate the sensitivity of our approach to the following parameters: number of particles *M*, the target ESS, the number of burn-in steps for each intermediate distribution, the learning rate η, and the mini-batch size. Each test is done by varying one parameter while keeping the others fixed at their default values.

**Run-time Environment.** Our experiments were executed on a laptop with an Intel i7 CPU and 8GB of ram, running Linux. For fair comparison, all code was single-threaded. Multithreading gives a considerable speedup when calculating the likelihood on large data sets but can introduce subtle complexities that are difficult to control for in tests of run-time performance.

**Models.** We evaluated our approach on 3 models:

- Bayesian linear regression with 6 parameters.
- Bayesian logistic regression with 10-dimensional observations and four classes. This model has 44 parameters.
- Bayesian Gaussian mixture model for two-dimensional data with 5 mixture components. This model has 25 parameters.

For each of the models, we generated data sets by sampling i.i.d. from the model’s conditional distribution with the parameters fixed. See Appendix B for a more detailed description of the models.

### 6.2. Distribution Shift

**Figure 1:**
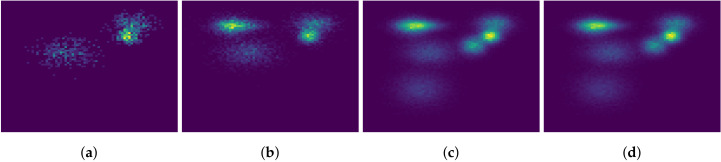
Histograms of non-stationary simulated data. (**a**) shows the first 1000 observations, (**b**) shows the next 9000 observations, (**c**) shows the last 90,000 observations, and (**d**) shows the total data set. In each of the three time phases, the data-generating distribution produces data with more clusters than before.

As described in Section 5, changes in the data-generating distribution should be detectable in the ML estimates. To investigate this, we generate simulated data with varying numbers of clusters. Histograms of the simulated data can be seen in Figure 1. The first 1000 observations were generated from 3 Gaussian distributions, the next 9000 observations were generated from 5 Gaussian distributions, including the 3 used to generate the first observations, and the remaining 90,000 observations were generated from 7 Gaussian distributions, including the previous 5. Some of the clusters overlap, so it is not immediately obvious from the histograms how many clusters there actually are.

We evaluate the effect of this type of distribution shift on SGAIS by estimating the ML online for Gaussian mixture models with 3, 5, and 7 mixture components. We then shuffle the data to enforce stationarity and estimate the ML for these three models on the shuffled data. If the final ML estimates for the in-order and shuffled data differ significantly then this may indicate that the particles are getting trapped in local modes before the change-points occur.

## 7. Results and Discussion

The log-ML typically grows linearly in the number of data points. For this reason, it is natural to measure errors in logZ/N rather than logZ. For each model, we measured the runtime performance of NS, AIS, and SGAIS for various data set sizes up to one million observations. Each of these data sets is taken as the first *N* observations of the largest data set. This allows SGAIS to leverage the previous ML estimates for online ML estimation. Due to computational constraints, we run NS and AIS only for data set sizes at logarithmically increasing intervals, while SGAIS naturally produces many more intermediate results in a single run. Since we found AIS to be much slower than NS, we only run AIS on data sets small enough to finish within 4000 s. We consider errors that are comparable to the discrepancy between NS and AIS acceptable.

### 7.1. Accuracy and Speed

#### 7.1.1. Linear Regression

**Figure 2:**
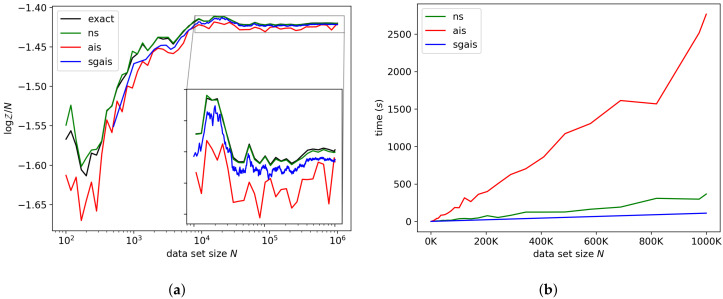
Linear regression model. (**a**) shows the accuracy of our marginal likelihood (ML) estimator compared to nested sampling (NS), annealed importance sampling (AIS), and the exact ML. (**b**) shows the run-time of each method. ns is nested sampling, ais is annealed importance sampling, and sgais is our stochastic gradient annealed importance sampling approach.

For the linear regression model, the exact ML is available analytically and is shown in Figure 2a for comparison. Each algorithm is able to produce accurate results for this model for all data set sizes. The final error of SGAIS on one million data points was only about 0.1%. For this model, our method achieved a speedup over NS by about a factor of 3.3, and a speedup over AIS by a factor of 24.9 on one million observations.

#### 7.1.2. Logistic Regression

**Figure 3:**
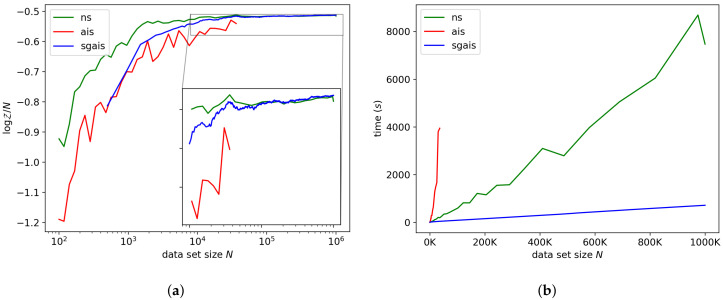
Logistic regression model. (**a**) shows our ML estimator compared to NS and AIS. (**b**) shows the run-time of each method. AIS was not run for larger data set sizes because each subsequent run would take more than 4000 s.

Figure 3a,b shows the log-ML estimates and run-time of each algorithm for the logistic regression model. For the largest data set, NS and SGAIS produced estimates that differed by roughly 0.6%, which is negligible. SGAIS was a factor 10.4 faster than the nested sampler on one million observations for this model.

#### 7.1.3. Gaussian Mixture Model

**Figure 4:**
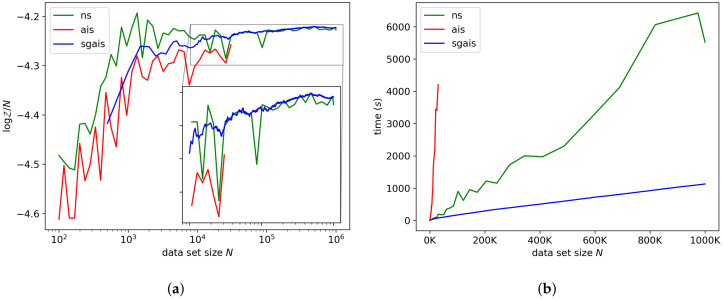
Gaussian mixture model. (**a**) shows our ML estimator compared to NS and AIS. (**b**) shows the run-time of each method.

Figure 4a,b shows the log-ML estimates and run-time of each algorithm for the Gaussian mixture model. The posterior distribution for this model is multimodal. Some modes are due to permutation symmetries; these modes do not have to be explored since each one contains the same information. There are also some local modes that do not necessarily capture meaningful information about the data; for example, fitting a single Gaussian to the whole data set may be a poor local optimum of the likelihood function. If an MCMC walker finds one of these modes, it can get trapped. However, we find that by Bayesian updating and annealing, the MCMC walkers tend to leave the poor local modes early on, before they become extremely peaked. The estimates produced by NS and SGAIS differed on the largest data set by roughly 0.1%. For this model, SGAIS was about a factor of 4.9 faster than the nested sampler for one million observations.

In all the above experiments, SGAIS converges to the same result as NS with a negligible error for large *N*.

### 7.2. Distribution Shift

**Figure 5:**
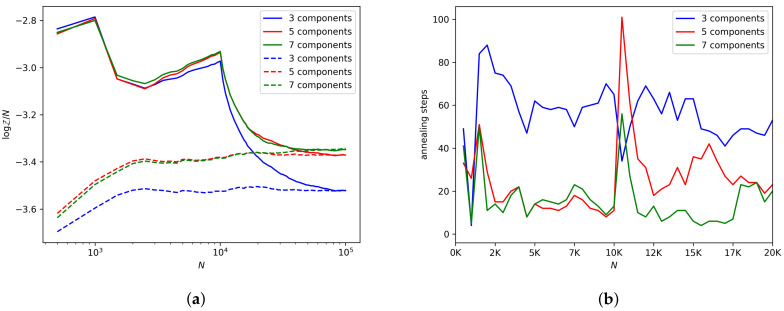
ML estimation under distribution shift. (**a**) shows the ML estimates for Gaussian mixture models with different numbers of mixture components. The solid lines are for the in-order data and the dashed lines are for the shuffled and therefore stationary data. (**b**) shows the number of annealing steps for the online ML estimates for the in-order data.

The log-ML estimates shown in Figure 5a display sharp changes at 1000 and 10,000 observations for the non-shuffled data. We are very clearly able to identify the position of the change-points just by looking at the resulting plot, without a priori assuming the existence or number of change-points. The numbers of annealing steps shown in Figure 5b exhibit spikes at the change-points and remain high once more clusters are added to the data than the model can describe.

The agreement of the final ML estimates between the shuffled and non-shuffled data suggests that these estimates can be trusted. The difference between the online and shuffled estimates is small enough to be able to distinguish between the three models. The 5- and 7-component models seem to describe the total data set better than the 3-component model, but the 5 and 7 models have similar values for their log-ML, presumably due to the overlapping clusters in the data set.

### 7.3. Sensitivity to Algorithm Parameters

The following results agreed closely with our expectations:

- Increasing the number of particles, *M*, results in higher accuracy and a longer running time without much effect on the number of annealing steps.
- A smaller number of burn-in SGHMC steps per intermediate distribution typically resulted in lower accuracy and a shorter run-time. A smaller number of burn-in steps also resulted in more annealing steps due to the slower equilibration.
- Larger mini-batch sizes typically result in higher accuracy but more computation per SGHMC step. Larger mini-batch sizes result in fewer Bayesian updating steps but require more annealing steps per new chunk of data. Mini-batch size would typically be chosen based on the hardware capabilities of the platform and the type of data under consideration.

We report on our more interesting findings below; plots of the results for each parameter investigated are given in Appendix C.

#### 7.3.1. Target ESS

**Figure 6:**
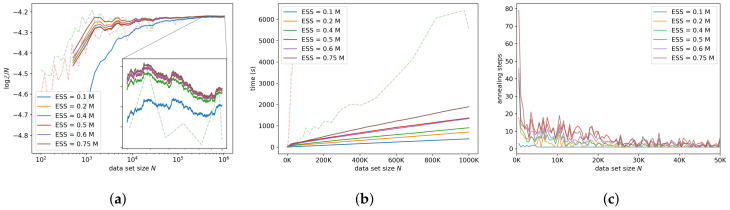
Sensitivity to the target effective sample size (ESS). (**a**) shows the log-ML estimates, (**b**) shows the run-time, and (**c**) shows the number of annealing steps for each chunk of data against the data set size until that chunk.

As expected, a higher target ESS tends to result in more annealing steps—see Figure 6a. Most of this work is done in the early stages of Bayesian updating. Note that since we used 10 particles, a target ESS of 0.1M=1 requires no annealing steps because ESS is bounded below by 1. No annealing results in high variance during the early stages of Bayesian updating, and adaptively annealing helps to reduce that variance, with only a small impact on the run-time. This illustrates the importance of the adaptive annealing schedule in our approach. Figure 6a,b indicates that our approach converges to the log-ML within acceptable accuracy within a reasonable time for a target ESS larger than 1. Even a small target ESS was good enough to match vanilla AIS, on average.

#### 7.3.2. Learning Rate

**Figure 7:**
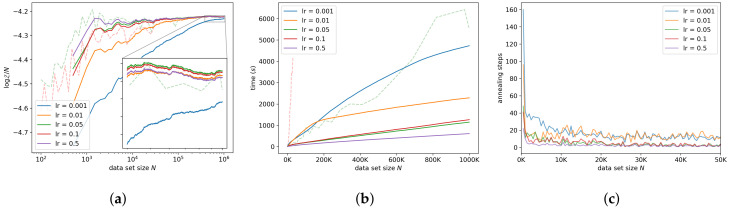
Sensitivity to the learning rate. Reported learning rates are per-observation learning rates, that is, η=lr/N. (**a**) shows the log-ML estimates, (**b**) shows the run-time, and (**c**) shows the number of annealing steps. For a learning rate of 0.01, the run-time shown in (**b**) displays a change in gradient near $10^{5}$ observations. This is the result of a reduced number of annealing steps but is not visible in (**c**) since we only show the number of annealing steps for up to $5\times 10^{4}$ observations.

Interestingly, smaller values of the learning rate tend to result in less accurate log-ML estimates over a longer time—see Figure 7a,b. We suspect this to be because a smaller learning rate does not allow the particle to move as far each step, resulting in a slower equilibration and requiring more annealing steps per observation. This effect can be seen in Figure 7c; the smaller learning rates appear to result in a larger number of annealing steps per observation. To further verify this, we investigated the interaction between the learning rate and the number of burn-in steps.

#### 7.3.3. Learning Rate and Burn-in

**Figure 8:**
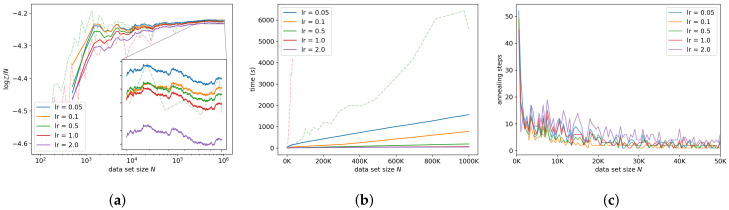
Sensitivity to the learning rate while keeping the product of the learning rate and the number of stochastic gradient Hamiltonian Monte Carlo (SGHMC) steps constant. Reported learning rates are per-observation learning rates, that is, η=lr/N. (**a**) shows the log-ML estimates, (**b**) shows the run time, and (**c**) shows the number of annealing steps.

We investigate the interaction between the number of SGHMC steps taken per intermediate distribution and the learning rate by varying the learning rate, while keeping the product of the learning rate and the number of SGHMC steps constant. Fewer burn-in steps (larger learning rate) tends to make the algorithm faster, but a smaller learning rate results in higher accuracy in the log-ML estimates, as seen in Figure 8a. The decrease in accuracy with a larger learning rate is presumably due to the discretization error in Equation (3). This is supported by Figure 8c: a larger learning rate requires a larger number of annealing steps to reach the target ESS. Figure 8a indicates that a per-observation learning rate of 1.0 can be used and still result in estimates of acceptable accuracy on data sets of one million observations. For a learning rate of 1.0, SGAIS achieved a speedup over NS by a factor of 83.

## 8. Materials and Methods

Code for this work was implemented using pytorch [20]. The code for our experiments is available at https://gitlab.com/pleased/sequential-evidence.

## 9. Conclusions

This paper introduced SGAIS, a novel algorithm for efficient large-scale ML estimation, by combining the essential ingredients of Bayesian updating, thermal annealing and data sub-sampling.We found that our SGAIS implementation was able to produce accurate ML estimates with a speedup over NS and AIS on simple models. Furthermore, since the marginal cost of updating the ML estimates when new data arrives does not depend on the number of previous data points, this approach may be effective for weighted model averaging in a setting where one periodically gets access to new data, such as in streaming applications.

We evaluated the sensitivity of our approach to the parameters and found that the log-ML estimates were robust to many different parameter choices while maintaining a speedup over NS and AIS for large data sets.

Potential future work may include further exploring the effects of stochastic gradients for ML calculation in the full SMC setting, including latent variable models, and with more general stochastic gradient MCMC algorithms such as Riemannian manifold SGHMC [17].

Lastly, we would like to point out that SGAIS naturally inherits some of the pitfalls of AIS and SGHMC such as sensitivity to phase changes, local modes, and the curse of dimensionality. Bayesian updating and adaptive scheduling does help to reduce some of the challenges that come with annealing, just as annealing helps to reduce problems arising from multi-modality, but to our knowledge, there is no way to completely remove these difficulties in general.

## Figures and Tables

**Figure A1:**
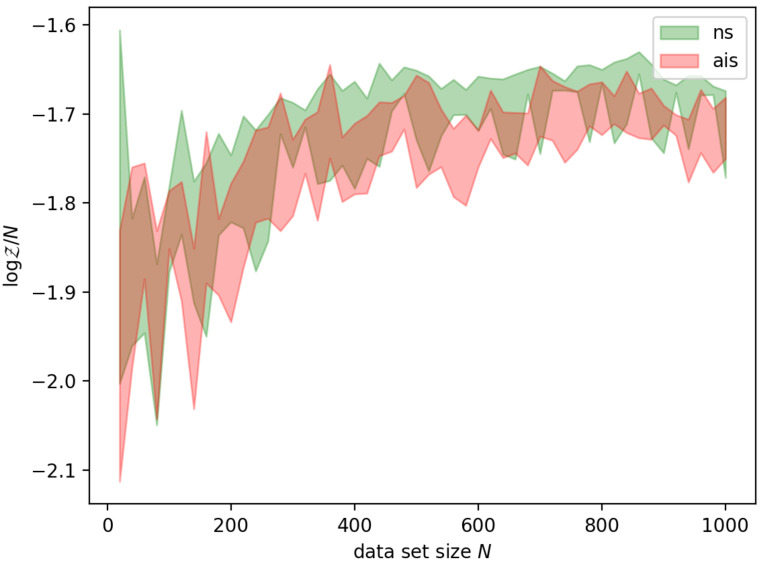
Comparison of NS and AIS with SGHMC kernels for a 1-dimensional Gaussian mixture model with 9 parameters (8 degrees of freedom). The shaded regions are the minimum and maximum of 5 independent runs of each algorithm.

**Figure A2:**
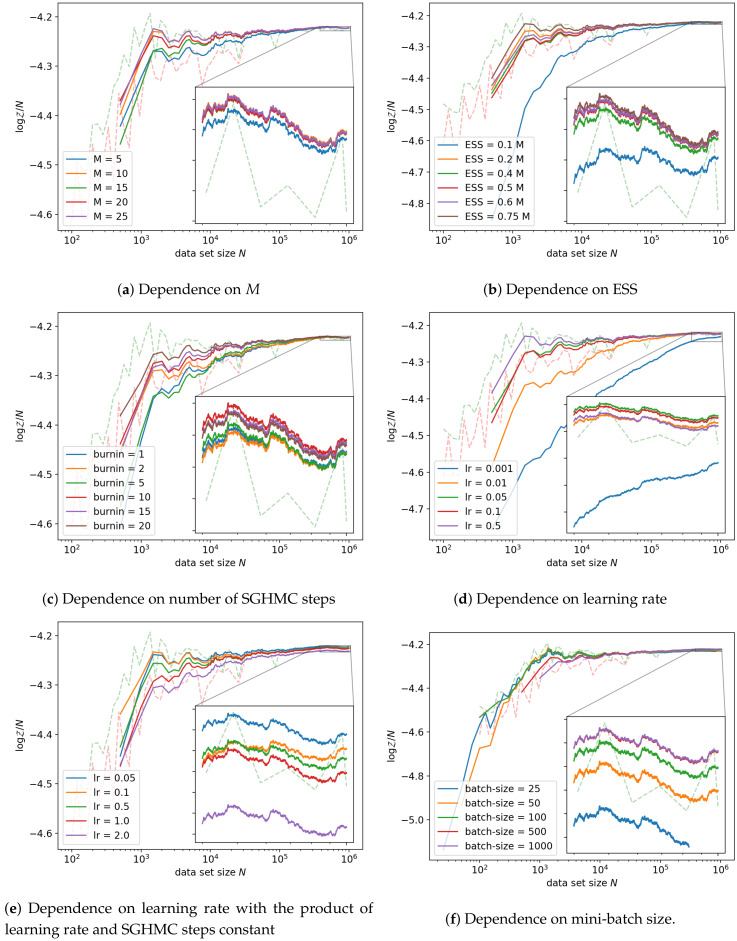
Log-ML sensitivity.

**Figure A3:**
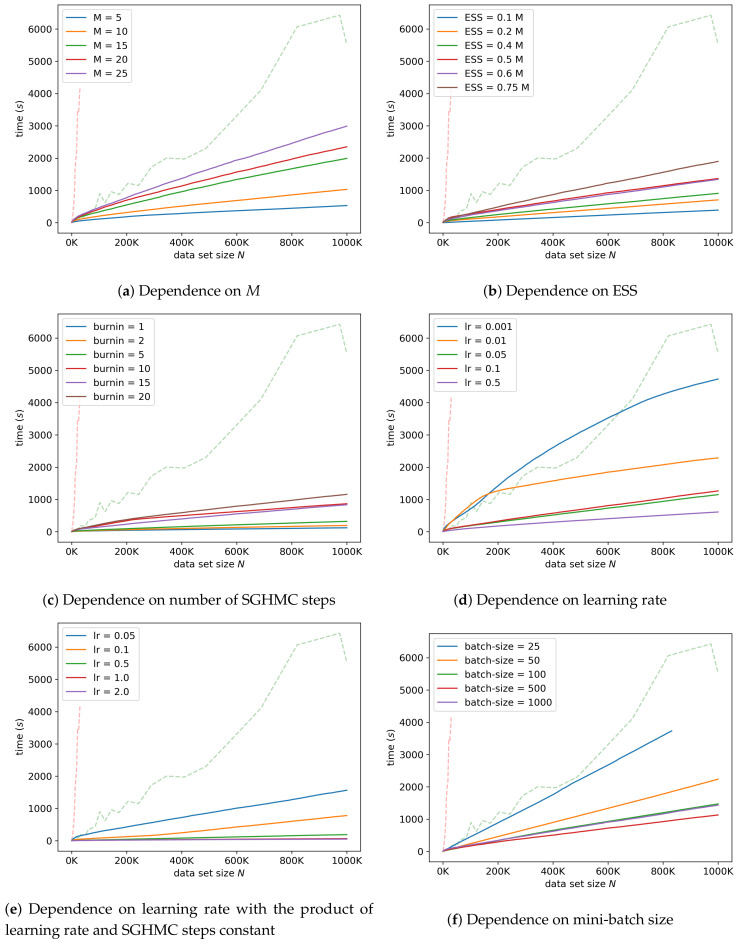
Running time sensitivity.

**Figure A4:**
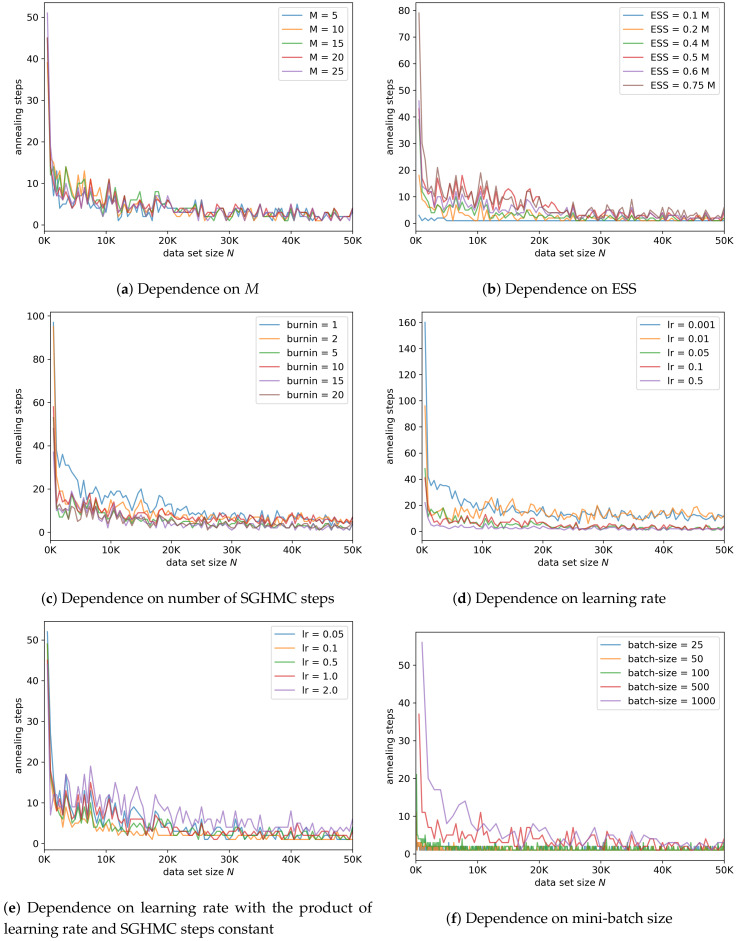
Sensitivity of number of annealing steps.

## Abbreviations

The following abbreviations are used in this manuscript:

AIS	Annealed importance sampling
ESS	Effective sample size
i.i.d	Independently and identically distributed
MCMC	Markov chain Monte Carlo
ML	Marginal likelihood
NS	Nested sampling
SGAIS	Stochastic gradient annealed importance sampling
SGHMC	Stochastic gradient Hamiltonian Monte Carlo
SMC	Sequential Monte Carlo

## Appendix A. Correctness of Nested Sampler and Annealed Importance Sampler

### Appendix A.1. Nested Sampler Implementation

Nested sampling requires one to sample from the prior under increasing likelihood constraints. Sampling under constraints is, in general, a difficult problem. This sampling can be accomplished by repeatedly sampling from the prior until the constraint is satisfied. This method of rejection sampling scales extremely poorly with both the size of the data set and the dimension of the parameter space and is not feasible on any but the smallest problems. Instead, the constrained sampling is typically implemented by duplicating a live particle, which is already within the constrained likelihood contour, and applying an MCMC kernel to the particle that leaves the prior invariant. While moving the particle with MCMC, care must be taken to avoid leaving the constrained region.

Our implementation of NS is based on SGHMC, simply because the code for SGHMC was already written. In order to sample under constraints, we use a similar strategy to Galilean Monte Carlo as described in [21], where the particle reflects off of the boundary of the likelihood contour by updating the momentum as follows:

Δv=−2(v·n)n.

Here, n is a unit vector parallel to the likelihood gradient $\nabla_{\theta }p\left(D|\theta \right)$. While it is not the focus of our paper, we note that we have not previously encountered the idea of applying SGHMC to sampling under constraints; our implementation allows a fairly direct approach to implementing NS in a variety of contexts, and the technique may also potentially be of value in other constrained sampling contexts.

We found that due to discretization error, the constrained sampler tends to undersample slightly at the boundaries of the constrained region; however, the undersampled volume is of the order of the learning rate η and so can be neglected for a sufficiently small learning rate.

### Appendix A.2. Test Results

If either of our NS and AIS implementations were incorrect, we expect that their ML estimates would disagree. If both implementations were incorrect, the probability that their errors would cancel is close to zero since NS and AIS work in completely different ways. Figure A1 shows the result of 5 independent runs of NS and AIS for data sets of varying sizes from 20 to 1000, in intervals of 20. The shaded regions are the minimum and maximum log-ML estimates for each run; these regions are the typical values produced by NS and AIS on these data sets. Since the typical results of NS and AIS tend to agree, it is safe to assume that they have been implemented correctly.

## Appendix B. Model Details

### Appendix B.1. Linear Regression

The data set consists of pairs (x,y) related by

$$y=w^{T}x+b+\epsilon ,$$

where ϵ c distributed with known variance $\sigma^{2}$. We do not assume any distribution over x as it always appears on the right-hand side of the conditional. The single-observation likelihood is

$$p\left(y|x,w,b\right)=\frac{1}{\sqrt{2\pi \sigma^{2}}}\exp\left(-\frac{{\left(y-w^{T}x-b\right)}^{2}}{2\sigma^{2}}\right).$$

Parameters are **w** and *b*, with standard Gaussian priors. ML can be calculated analytically for this model. We used 5 dimensional vectors x; this model therefore has 6 parameters.

### Appendix B.2. Logistic Regression

The data set consists of pairs (x,y), where x is an observation vector that is assigned a class label y∈{1,…,K}. The labels have a discrete distribution with probabilities given by the softmax function of an affine transform of the observations

$$p\left(y|x,\theta \right)=\frac{\exp(w_{y}^{T}x+b_{y})}{\sum_{k}\exp\left(w_{k}^{T}x+b_{k}\right)}.$$

Parameters are $\theta =(w_{1:K},b_{1:K})$, with standard Gaussian priors. Again, we do not assume any distribution over x as it always appears on the right-hand side of the conditional. We used 10 dimensional vectors x with 4 classes; this model has 44 parameters.

### Appendix B.3. Gaussian Mixture Model

The data are modeled by a mixture of *d*-dimensional multivariate Gaussian distributions with diagonal covariance matrices. Mixture weights, means, and variances are treated as parameters. This type of model is often treated as a latent variable model, where the mixture component assignments of each data point are the latent variables. Here, we marginalize out the latent variables to obtain the following conditional distribution:

$$p\left(y|\theta \right)=\sum_{k=1}^{K}\beta_{k}\prod_{j=1}^{d}\frac{1}{\sqrt{2\pi \sigma_{k,j}^{2}}}\exp\left(-\frac{{\left(y_{j}-\mu_{k,j}\right)}^{2}}{2\sigma_{k,j}^{2}}\right),$$

$$\theta =(\beta_{1:K},\mu_{1:K,1:d},\sigma_{1:K,1:d}^{2}).$$

Mixture weights $\beta_{1:K}$ are modeled by a Dirichlet prior with α=1; means $\mu_{k,j}$ are modeled conditionally given the variances by Gaussian priors, centered around zero with variance $4\sigma_{k,j}^{2}$; variances $\sigma_{k,j}^{2}$ are modeled by inverse gamma priors with shape and scale parameters equal to 1. We used 5 Gaussian components, and observations were 2-dimensional; this model has 25 parameters with 24 degrees of freedom. We use this model for our sensitivity tests, since it has the most complex structure.
